# Клинический полиморфизм первичного гиперпаратиреоза у детей

**DOI:** 10.14341/probl13489

**Published:** 2025-05-20

**Authors:** А. Р. Бенина, А. А. Колодкина, Н. Ю. Калинченко, О. Б. Безлепкина

**Affiliations:** Национальный медицинский исследовательский центр эндокринологии; Национальный медицинский исследовательский центр эндокринологии; Национальный медицинский исследовательский центр эндокринологии; Национальный медицинский исследовательский центр эндокринологии

**Keywords:** первичный гиперпаратиреоз, дети, гиперкальциемия, костные деформации, мочекаменная болезнь, неврологические симптомы

## Abstract

**ОБОСНОВАНИЕ:**

ОБОСНОВАНИЕ. Первичный гиперпаратиреоз (ПГПТ) у детей встречается достаточно редко, распространенность составляет 2–5 случаев на 100 000 детского населения. Гиперкальциемия при ПГПТ оказывает негативное влияние на органы желудочно-кишечного тракта, мочевыделительную, опорно-двигательную и нервную системы. Своевременная диагностика заболевания у детей затруднена в связи с редкой встречаемостью и разнообразием клинических симптомов.

**ЦЕЛЬ:**

ЦЕЛЬ. Изучить клинические проявления первичного гиперпаратиреоза у детей в зависимости от степени гиперкальциемии.

**МАТЕРИАЛЫ И МЕТОДЫ:**

МАТЕРИАЛЫ И МЕТОДЫ. Ретроспективное с проспективным компонентом наблюдательное исследование 50 детей с первичным гиперпаратиреозом. Всем пациентам проведено комплексное лабораторно-инструментальное исследование в Институте детской эндокринологии ФГБУ «НМИЦ эндокринологии» Минздрава России в период 2014–2023 гг.

**РЕЗУЛЬТАТЫ:**

РЕЗУЛЬТАТЫ. Клинические проявления первичного гиперпаратиреоза у детей очень разнообразны. Слабость и утомляемость наблюдались у 36% пациентов (ДИ (23; 51)). Частыми проявлениями со стороны ЖКТ были тошнота — у 20% (ДИ (10; 34)), гастрит — у 38% (ДИ (25; 53)), дуоденогастральный рефлюкс — у 24% (ДИ (13; 38)). Гиперкальциурия выявлена у 64% пациентов (ДИ (49; 77)), мочекаменная болезнь (МКБ) — у 36% (ДИ (23; 51)). Жалобы на боли в ногах наблюдались у 24% пациентов (ДИ (13; 38)), деформация нижних конечностей была у 20% (ДИ (10; 34)). Низкотравматические переломы в анамнезе имели 16% пациентов (ДИ (7; 29)). Медиана возраста появления первых клинических симптомов ПГПТ у детей составила 13,7 года [10,6; 15,2]. На момент диагностики заболевания 12 пациентов (24%) не имели жалоб и были обследованы в связи со случайно выявленной гиперкальциемией (n=3), гиперкальциурией (n=1), выявленными образованиями околощитовидных желез (ОЩЖ) по данным УЗИ (n=5), наличием отягощенного наследственного анамнеза по синдрому множественных эндокринных неоплазий 1 типа (n=3). Для выявления связи между клиническими проявлениями ПГПТ и уровнем кальция крови все пациенты были разделены на 3 группы в зависимости от уровня гиперкальциемии: легкая степень гиперкальциемии — 29 пациентов, умеренная — 16, тяжелая — 5. По результатам исследования статистически значимой связи между наличием отдельных клинических проявлений ПГПТ и степенью гиперкальциемии выявлено не было, однако отмечена статистическая тенденция между наличием отдельных клинических проявлений заболевания (гиперкальциурия, снижение массы тела, рвота, боль в животе, запоры, эзофагит, боль в ребрах, нарушение походки) и уровнем кальция крови, а также была выявлена положительная ассоциация между гиперкальциурией и гиперкальциемией. Кроме того, отмечено, что у пациентов с тяжелой гиперкальциемией количество клинических признаков значимо выше, чем у пациентов с легкой или умеренной степенью гиперкальциемии.

**ЗАКЛЮЧЕНИЕ:**

ЗАКЛЮЧЕНИЕ. Проведенное исследование демонстрирует многообразие клинических проявлений ПГПТ, по поводу которых дети могут наблюдаться у врачей разных специальностей — педиатров, гастроэнтерологов, нефрологов, неврологов, травматологов-ортопедов. При наличии тошноты, утомляемости, боли в ногах, деформации нижних конечностей, низкотравматических переломов, мочекаменной болезни, гастрита необходимо исследовать уровень кальция в крови у детей.

## Abstract (en)

**BACKGROUND:**

BACKGROUND: Primary hyperparathyroidism (PHPT) in children is quite rare, with a prevalence of 2–5 cases per 100,000 children. Hypercalcemia in PHPT has a negative impact on the gastrointestinal tract, urinary, musculoskeletal and nervous systems. Timely diagnosis of the disease in children is difficult due to its rare occurrence and variety of clinical symptoms.

**AIM:**

AIM: To study clinical manifestations of primary hyperparathyroidism in children depending on the degree of hypercalcemia.

**MATERIALS AND METHODS:**

MATERIALS AND METHODS: Retrospective observational study with a prospective component 50 patients with primary hyperparathyroidism. All patients underwent a comprehensive laboratory and instrumental study at the Institute of Pediatric Endocrinology, Endocrinology Research Center of Russia in the period 2014–2023.

**RESULTS:**

RESULTS: The clinical manifestations of primary hyperparathyroidism in children are very diverse. Weakness and fatigue were observed in 36% of patients (CI (23; 51)). Frequent manifestations of the gastrointestinal tract were nausea — in 20% (CI (10; 34)), gastritis — in 38%, (CI (25; 53)), duodenogastric reflux - in 24%, CI (13; 38). Hypercalciuria was detected in 64% of patients (CI (49; 77)), urolithiasis - in 36% (CI (23; 51). Complaints of leg pain were observed in 24% of patients (CI (13; 38)) deformity of the lower extremities was present in 20% (CI (10; 34)). 16% of patients had a history of low-traumatic fractures (CI (7; 29)). The median age of onset of the first clinical symptoms of PHPT in children was 13.7 years [10.6; 15.2]. At the time of diagnosis of the disease, 12 patients (24%) had no complaints and were examined due to incidentally detected hypercalcemia (n=3), hypercalciuria (n=1), and detected formations of the parathyroid gland according to ultrasound (n=5), with a family history of multiple endocrine neoplasia syndrome type 1 (n=3). To identify the relationship between the clinical manifestations of PHPT and blood calcium levels, all patients were divided into 3 groups depending on the level of hypercalcemia: mild hypercalcemia — 29 patients, moderate — 16, severe — 5. According to the results of the study, a statistically significant relationship between the presence of individual clinical manifestations of PHPT and the degree of hypercalcemia was not identified, however, a statistical tendency was noted between the presence of individual symptoms of the disease (hypercalciuria, weight loss, vomiting, pain in abdomen, constipation, esophagitis, rib pain, gait disturbance) and blood calcium levels, and a positive association was found between hypercalciuria and hypercalcemia. In addition, it was noted that in patients with severe hypercalcemia, the number of clinical signs is significantly higher than in patients with mild or moderate hypercalcemia.

**CONCLUSION:**

CONCLUSION: The study demonstrates the variety of clinical manifestations of PHPT, for which doctors of various specialties — pediatricians, gastroenterologists, nephrologists, neurologists, orthopedic traumatologists, can observe children. In the presence of symptoms and diseases such as fatigue, nausea, pain in the legs, deformation of the lower extremities, low-traumatic fractures, urolithiasis, gastritis, it is necessary to examine the level of calcium in the blood in children.

## ОБОСНОВАНИЕ

Первичный гиперпаратиреоз характеризуется избыточной секрецией паратгормона (ПТГ) при верхне-нормальном или повышенном уровне кальция крови [1]. Причиной ПГПТ является изолированное или множественное поражение околощитовидных желез (ОЩЖ), морфологически представляющее собой гиперплазию, аденому, атипическую аденому или карциному ОЩЖ [1].

По данным зарубежных исследований, распространенность ПГПТ составляет 0,84–1% [2][3], заболевание значительно чаще встречается у взрослых, чем у детей. В России не проводилось крупных эпидемиологических исследований по изучению распространенности ПГПТ. По данным Российского регистра первичного гиперпаратиреоза, выявлена низкая распространенность (1,3 случая на 100 000 взрослого населения) [4]. По данным зарубежных исследований, распространенность заболевания у детей составляет 2–5:100 000 [5–7]. Отечественные публикации, касающиеся ПГПТ у детей, единичны и в большинстве своем посвящены описанию клинических случаев [8–10]; в частности в работах А.В. Гостимского представлен опыт хирургического лечения ПГПТ у детей [11][12].

У детей своевременная диагностика ПГПТ затруднена в связи с неспецифичными клиническими проявлениями в дебюте заболевания, а в некоторых случаях — бессимптомным течением (до 10–30% случаев) [6][7]. У детей в рутинной клинической практике уровень кальция крови исследуется редко.

Выделяют три степени гиперкальциемии: легкую, умеренную и тяжелую [1][13]. Гиперкальциемия приводит к поражению разных органов и систем, в том числе ЖКТ, мочевыделительной, костно-мышечной и нервной систем [1].

В зарубежных исследованиях у взрослых пациентов не было выявлено статистически значимой связи между тяжестью гиперкальциемии и наличием симптомов ПГПТ [14][15], у детей аналогичных исследований не проводилось.

## ЦЕЛЬ ИССЛЕДОВАНИЯ

Изучить клинические проявления первичного гиперпаратиреоза у детей в зависимости от степени гиперкальциемии.

## МАТЕРИАЛЫ И МЕТОДЫ

## Место и время проведения исследования

Место проведения: Институт детской эндокринологии ФГБУ «НМИЦ эндокринологии» Минздрава России.

Время исследования: сбор данных для исследования проводился в период с сентября 2014-го по январь 2023 гг.

## Изучаемые популяции

Популяция: в исследование включено 50 пациентов (23 мальчика, 27 девочек) в возрасте от 10 до 17 лет с диагнозом: «Первичный гиперпаратиреоз».

Критерии включения: пациенты с гиперкальциемией на фоне высокого ПТГ, наличие письменного информированного согласия родителей или законного опекуна пациента.

Критерии исключения: пациенты с хронической почечной недостаточностью, с вторичным и третичным гиперпаратиреозом.

## Способ формирования выборки из изучаемой популяции

Сплошной способ формирования выборки.

## Дизайн исследования

Одноцентровое одновыборочное ретроспективное исследование с проспективным компонентом.

## Методы

Протокол исследования включал в себя оценку жалоб, сбор анамнеза жизни и заболевания, клинический осмотр с оценкой антропометрических данных и наличия костных деформаций.

Гормональные и биохимические исследования проводились в клинико-диагностической лаборатории ФГБУ «НМИЦ эндокринологии» Минздрава России и включали в себя оценку уровня ПТГ (норма 15–65 пг/мл), общего кальция (норма 2,29–2,63 ммоль/л), ионизированного кальция (1,03–1,29 ммоль/л) в сыворотке крови; исследование уровня кальция в разовой и/или суточной порциях мочи. Критерием гиперкальциурии считалось повышение уровня кальция в моче выше референсных значений 1,7–5,3 ммоль/л для разовой порции мочи и 2,5–8 ммоль/сут. для суточной порции мочи.

Ультразвуковое исследование (УЗИ) проводилось на ультразвуковом сканере (Voluson E8, GE Healthcare, Австрия) с использованием линейного датчика с частотой 10–12 Мгц и включало в себя исследование ОЩЖ без предварительной подготовки пациента в положении лежа на спине с запрокинутой головой. В случае выявления образования ОЩЖ по своей структуре оно визуализировалось как гомогенное, гипоэхогенное или изоэхогенное и гиперваскулярное, чаще округлой/овальной формы. Для диагностики осложнений ПГПТ в виде мочекаменной (МКБ) и желчнокаменной болезней (ЖКБ) проводилось УЗИ почек и органов брюшной полости.

Оценка костных деформаций нижних конечностей проводилась с помощью рентгенографии в прямой проекции.

Для диагностики осложнений ЖКТ в виде гастрита, эзофагита и рефлюкса проводилась фиброгастродуоденоскопия (ФГДС) с последующей консультацией гастроэнтеролога.

Для оценки связи между клиникой ПГПТ и уровнем кальция крови пациенты были распределены на 3 группы, в зависимости от степени гиперкальциемии по уровню общего кальция крови: 1 группа — легкая гиперкальциемия (<3,0 ммоль/л), 2 группа — умеренная гиперкальциемия (3,0–3,5 ммоль/л), 3 группа — тяжелая гиперкальциемия (>3,5 ммоль/л).

## Статистический анализ

Расчет данных производился с помощью статистического пакета Statistica 13 (StatSoft inc., США), MS Exel 2016 (Microsoft, США). Количественные результаты представлены в виде медианы (Ме) и квартилей [ Q1; Q3], соответствующих 25 и 75 перцентилям. Качественные данные представлены в виде абсолютных (n) и относительных (%) частот, их 95% доверительный интервал (ДИ) рассчитан с помощью метода Клоппера-Пирсона. Для выявления статистически значимых различий между количественными признаками трех независимых групп использован критерий Краскела-Уоллиса, для качественных признаков — критерий Фримена-Холтона. Корреляционный анализ проводился методом ранговой корреляции по Спирмену. Для выявления ассоциации наиболее значимых клинических симптомов в зависимости от уровня кальция крови проведен многофакторный линейный регрессионный анализ. Критический уровень значимости различий принимали р≤0,05. С целью решения проблемы множественных сравнений применялся p-value, скорректированный поправкой Бонферрони. Статистической тенденцией считали различия при р-value от поправки Бонферрони до 0,05.

## Этическая экспертиза

Проведение исследования одобрено локальным этическим комитетом ФГБУ «НМИЦ эндокринологии» Минздрава России 12.10.2022 г., протокол №18.

## РЕЗУЛЬТАТЫ

В исследование включено 50 детей с ПГПТ (23 мальчика и 27 девочек), медиана возраста 15,7 года [ 13,0; 16,8]. Анализ анамнестических данных показал, что возраст появления первых клинических симптомов ПГПТ у детей составил 13,7 года [ 10,6; 15,2].

Слабость и утомляемость были самыми частыми симптомами и встречались у 36% пациентов (n=18, ДИ (23; 51)). Жалобами со стороны ЖКТ были тошнота у 20% пациентов (n=10, ДИ (10; 34)), боль в животе — у 14% (n=7, ДИ (6; 27)), снижение массы тела — у 12% пациентов (n=6, ДИ (5; 24)), запоры — у 8% (n=4, ДИ (2; 19)). Со стороны опорно-двигательной системы пациенты жаловались на боли в ногах — 24% (n=12, ДИ (13; 38)), у 10 из них имелись деформации нижних конечностей, а низкотравматические переломы были у 8 пациентов.

Треть пациентов, прежде чем им был диагностирован ПГПТ, наблюдались у врачей других специальностей. Под наблюдением гастроэнтеролога находилось 8 детей (16%, ДИ (7; 29), 6 из них по поводу гастрита (3 с сопутствующим рефлюкс-эзофагитом) и 2 — с ЖКБ. Стационарное лечение по поводу панкреатита в анамнезе имелось у 4 пациентов (8%, ДИ (2; 19)). У нефролога с МКБ наблюдались 4 пациента (8%, ДИ (2; 19)), 2 пациента (4%, ДИ (0; 14)) — с болезненностью при мочеиспускании, обусловленной гиперкальциурией.

Срок от появления первых симптомов до постановки диагноза ПГПТ составил 1 год [ 0,34; 2,2].

На момент постановки диагноза медиана уровня ПТГ составила 149 пг/мл [ 87; 556], общего кальция — 2,98 ммоль/л [ 2,7; 3,2], ионизованного кальция — 1,38 ммоль/л [ 1,3; 1,5]. Гиперкальциурия наблюдалась у 32 пациентов (64%, ДИ (49;77)), у 18 из них (36%, ДИ (23; 51)) диагностирована МКБ.

Для оценки связи между клиническими проявлениями ПГПТ и уровнем кальция крови пациенты были разделены на 3 группы в зависимости от степени тяжести гиперкальциемии: группа с легкой гиперкальциемией состояла из 29 детей, группа с умеренной гиперкальциемией из 16 детей, группа с тяжелой гиперкальциемией включала в себя 5 детей.

В таблице 1 представлены уровни ПТГ и кальция крови у детей трех групп. Примененный для анализа критерий Краскела-Уоллиса показал достоверные различия значений ПТГ и кальция (общего и ионизированного) между пациентами трех групп. Обращает на себя внимание, что у детей с тяжелой гиперкальциемией уровень ПТГ был значительно выше, чем у детей с легкой и умеренной гиперкальциемией. Корреляционный анализ выявил статистически значимую положительную взаимосвязь ПТГ с кальцием общим (r=0,51, р=0,0002) и кальцием ионизированным (r=0,74, р<0,001, метод ранговой корреляции Спирмена, р0=0,017 после поправки Бонферрони).

**Table:** Таблица 1. Уровни ПТГ и кальция у детей с первичным гиперпаратиреозом Примечание: пороговый Р=0,017 (после применения поправки Бонферрони).

Лабораторные показатели	Степень гиперкальциемии	Критерий Краскела-Уоллиса
Легкая (n=29)	Умеренная (n=16)	Тяжелая (n=5)
ПТГ (пг/мл)	103 [ 81; 152]	231 [ 143,9; 665]	1262 [ 566,3; 2012]	0,0005
Кальций общий (ммоль/л)	2,79 [ 2,68; 2,9]	3,2 [ 3; 3,28]	3,87 [ 3,83; 4,17]	<0,001
Кальций ионизированный (ммоль/л)	1,3 [ 1,2; 1,38]	1,45 [ 1,4; 1,5]	1,75 [ 1,7; 2]	<0,001

Была проведена оценка наиболее часто встречающихся симптомов и заболеваний, характеризующих поражение различных систем и органов у детей с ПГПТ. Результаты представлены в таблице 2.

**Table:** Таблица 2. Клинические проявления у детей с первичным гиперпаратиреозом Примечание: пороговый P=0,0015 (после применения поправки Бонферрони).

Клинические проявления ПГПТ	Степень гиперкальциемии	Критерий Фримена-Холтона (Р)
Легкая (n=29)	Умеренная (n=16)	Тяжелая (n=5)
Наличие признака
Мочевыделительная система
гиперкальциурия	13	14	5	0,003
мочекаменная болезнь	9	7	2	0,690
диффузные изменения почек	3	3	2	0,200
боль при мочеиспускании	1	1	0	0,900
полиурия/полидипсия	0	0	2	0,080
Желудочно-кишечный тракт
гастрит	10	6	3	0,620
дуоденогастральный рефлюкс	7	3	2	0,700
тошнота	5	2	3	0,070
снижение массы тела	4	0	2	0,045
рвота	2	0	3	0,003
боль в животе	1	3	3	0,003
запоры	1	0	3	0,002
эзофагит	0	2	2	0,007
панкреатит	1	2	1	0,200
желчнокаменная болезнь	0	2	1	0,080
Опорно-двигательная система
боль в ногах	6	3	3	0,200
вальгусная деформация нижних конечностей	6	2	2	0,090
переломы в анамнезе	4	2	2	0,300
мышечная гипотония	3	1	1	0,600
нарушение походки	3	1	3	0,020
деформация грудной клетки	3	0	1	0,300
сколиоз	3	1	1	0,600
лордоз	0	2	0	0,200
боль в спине	2	2	0	0,700
боль в ребрах	1	0	2	0,020
боль в пальцах рук	0	1	0	0,400
Нервная система
слабость/утомляемость	10	5	3	0,500
головная боль	3	3	1	0,600
эмоциональная лабильность	0	1	1	0,070
гиперестезия	0	1	1	0,070
депрессия и галлюцинации	0	1	0	0,400
Другие симптомы
чувство сдавления в области шеи и кашель	1	1	0	0,900
субфебрильная температура	0	1	1	0,070

Гиперкальциурия встречалась у пациентов во всех трех группах: у 45% пациентов с легкой гиперкальциемией, у 87,5% пациентов с умеренной и у 100% пациентов с тяжелой гиперкальциемией. Мочекаменная болезнь также встречалась у пациентов во всех трех группах, однако тяжесть симптоматики была различной, так, например, у 1 пациента с умеренной гиперкальциемией МКБ манифестировала с почечной колики, что в дальнейшем потребовало проведение дистанционной литотрипсии.

У каждого 4-го ребенка были жалобы на периодическую тошноту, независимо от степени гиперкальциемии: у 2 пациентов (6%, ДИ (0; 23)) с легкой гиперкальциемией и у 3 пациентов (60%, ДИ (15; 95)) с тяжелой гиперкальциемией часто тошнота сопровождалась рвотой. У 12% пациентов (n=6, ДИ (4; 24)) к моменту диагностики заболевания имелось значительное снижение массы тела, у 1 пациентки 16 лет с тяжелой гиперкальциемией снижение веса составило 23 кг.

Вальгусная деформация нижних конечностей наблюдалась у каждого 5-го пациента (n=10, 20%), независимо от степени гиперкальциемии.

Слабость, утомляемость и головная боль также часто встречались у детей с ПГПТ независимо от степени гиперкальциемии.

На момент госпитализации 12 пациентов (24%, ДИ (13; 38)) не имели жалоб. Поводом для их обследования являлись: отягощенный наследственный анамнез по синдрому множественных эндокринных неоплазий 1 типа (n=3), случайно выявленная гиперкальциемия (n=3), гиперкальциурия (n=1), образование ОЩЖ по данным УЗИ (n=5). Среди них у 8 пациентов была легкая степень гиперкальциемии, у 4 — умеренная. При дальнейшем обследовании у 4 пациентов диагностированы осложнения, среди которых МКБ (n=1), деформации костей (n=2), а у 1 пациента наблюдалось сочетание МКБ и гастрита.

Несмотря на различия в тяжести проявлений клинических симптомов у отдельных пациентов, проведенный сравнительный анализ между 3 группами не выявил значимой связи между степенью гиперкальциемии и отдельными симптомами ПГПТ (табл. 2). Однако при повышении уровня кальция крови наблюдается увеличение некоторых клинических симптомов (гиперкальциурия, снижение массы тела, рвота, боль в животе, запоры, эзофагит, боль в ребрах, нарушение походки) на уровне статистической тенденции.

Для поиска сочетания разных признаков и симптомов в ассоциации с гиперкальциемией был проведен многофакторный регрессионный анализ. Были определены наиболее часто встречающиеся при ПГПТ клинические признаки: утомляемость, тошнота, гастрит, рефлюкс, боль в ногах, деформация нижних конечностей, низкотравматические переломы, гиперкальциурия, МКБ. При анализе выявлено, что только гиперкальциурия обладает положительной ассоциацией с гиперкальциемией (р=0,0024).

Для сравнения групп по количеству клинических признаков проведен суммарный подсчет симптомов и заболеваний у каждого пациента. Так, у пациентов с легкой гиперкальциемией наблюдалось от 1 до 9 признаков, в группе с умеренной гиперкальциемией — от 1 до 12, с тяжелой — от 8 до 16 признаков. Сравнительный анализ в трех группах показал, что количество клинических признаков при тяжелой степени гиперкальциемии было статистически значимо выше, чем при легкой и умеренной степени гииперкальциемии (табл. 3).

**Table:** Таблица 3. Сравнение детей в трех группах по совокупности клинических проявлений

Степень гиперкальциемии	Легкая (n=29)	Умеренная (n=16)	Тяжелая (n=5)	Критерий Краскела-Уоллиса (Р)
Количество симптомов	4 [ 2; 5]	3 [ 2; 6]	10 [ 9; 10]	0,0019 Р 1-2=1,0000 Р 1-3=0,0010 Р 2-3=0,0120

## ОБСУЖДЕНИЕ

Гиперкальциемия оказывает негативное влияние на работу органов ЖКТ, мочевыделительной, опорно-двигательной и нервной систем, обуславливая многообразный характер клинической картины у детей [16]. Зачастую пациенты длительное время наблюдаются у врачей разных специальностей (нефрологи, гастроэнтерологи, неврологи и др.), прежде чем им будет поставлен верный диагноз.

По данным зарубежной литературы, на момент диагностики заболевания около 70–90% детей имеют жалобы и клинику, ассоциированные с ПГПТ [5, 6]. В нашем исследовании 76% детей имели клинические признаки заболевания и диагноз ПГПТ у половины пациентов был установлен в течение 1 года от появления первых симптомов, у 6 пациентов — в течение 2 лет, у остальных пациентов ПГПТ был диагностирован через 2–4 года.

В работах J. Kollars с соавт. и W. Wang с соавт. возраст детей, при котором был установлен ПГПТ, варьировал от 5 до 18 лет, в нашем исследовании — от 10 до 17 лет [5][6].

В таблице 4 представлены обобщенные данные зарубежных исследований по клиническим проявлениям ПГПТ у детей совместно с нашими результатами. Согласно Европейскому урологическому консенсусу 2023 г. ПГПТ является причиной образования почечных камней в 5% случаев, среди них около 20% состоят из оксалата или фосфата кальция [17]. По данным Velásquez-Forero с соавт., МКБ у детей встречается редко и составляет 0,01–0,03% [18]. Как видно из таблицы 4, МКБ встречалась в 26–50% случаев по результатам зарубежных исследований, в нашей работе МКБ диагностирована у 36% пациентов (ДИ (23; 51)) [5][6][19–21].

**Table:** Таблица 4. Клинические проявления первичного гиперпаратиреоза у детей по данным крупных исследований Примечания: прочерк — признак не оценивался.

Страна исследования	Hiya Boro, et al. (Индия) [19]	Wenbo Wang, et al. (Китай) [6]	Yasmine El, Allali, et al. (Франция) [20]	Josh Kollars, et al. (США) [7]	Thomas Szabo Yamashita, et al. (США) [21]	Результаты проведенного исследования (Россия)
Число пациентов в исследовании (n)	10	59	39	52	66	50
Период исследования	2018–2019	1975–2015	1998–2018	1970–2000	1994–2020	2014–2023
Число пациентов
Клинические проявления	n (%)	n (%)	n (%)	n (%)	n (%)	n (%)
Гиперкальциурия	-	45 (81,8%)	22 (63%)	-	-	32 (64%)
Мочекаменная болезнь	5 (50%)	23 (39%)	10 (26%)	17 (33%)	22 (35%)	18 (36%)
Гиперкальциемический криз	-	6 (10,2%)	-	-	-	-
Полиурия	-	-	7 (18%)	5 (10%)	-	2 (4%)
Симптомы рефлюкса	4 (40%)	-	-	-	-	12 (24%)
Запоры	5 (50%)	-	-	5 (10%)	5 (8%)	4 (8%)
Тошнота	-	-	-	15 (29%)	6 (9%)	10 (20%)
Боль в животе	-	-	-	13 (25%)	7 (10%)	7 (14%)
Снижение массы тела	-	-	12 (31%)	6 (11%)	-	6 (12%)
Панкреатит	3 (30%)	4 (6,8%)	-	4 (7%)	3 (3%)	4 (8%)
Мышечная боль	9 (90%)	-	3 (8%)	-	4 (6%)	-
Вальгусная деформация нижних конечностей	5 (50%)	-	-	-	-	10 (20%)
Переломы конечностей	3 (30%)	20 (33,9 %)	3 (8%)	5 (10%)	13 (22%)	8 (16%)
Перелом позвоночника	4 (40%)	-	-	-	-	-
Поднадкостничная резорбция пальцев	8 (80%)	-	-	-	-	-
Остеопения/ остеомаляция	9 (90%)	27 (45,8%)	-	-	4 (6%)	-
Утомляемость/усталость	7 (70%)	-	-	18 (35%)	16 (24%)	18 (36%)
Бессимптомное течение	0	1 (1,7%)	6 (15%)	-	-	12 (24%)

Симптомы диспепсии могут быть первыми проявлениями ПГПТ у детей, которые встречаются в 19,5–87,5% случаев заболевания [14][20][21]. В проведенном исследовании у детей наблюдались тошнота, рвота и боль в животе в 20, 10 и 14% случаев соответственно, что сопоставимо с данными зарубежных исследований [5][6][19–21].

Распространенность панкреатита среди больных с ПГПТ варьирует от 3 до 15% [24]. Причиной для его развития является повышение уровня кальция в панкреатическом соке, что приводит к ускорению активации трипсиногена в трипсин, обуславливая повреждение органа [23][24]. В нашей группе 4 пациента (8%; ДИ (2; 19)) наблюдались по поводу панкреатита, прежде чем им был диагностирован ПГПТ, что сопоставимо с данными других исследований [5][6][19].

При ПГПТ причиной поражения костной системы является усиление работы остеокластов под действием высоких концентраций ПТГ и как следствие — повышение костной резорбции [27]. В результате происходит снижение минеральной плотности костной ткани, что приводит к деформациям и низкотравматическим переломам [27]. Механизм возникновения вальгусной деформации при ПГПТ до конца не изучен. Ряд авторов предполагают, что изменения возникают от прямого воздействия высоких концентраций ПТГ на ростовую пластину [28]. В работе Nipun Lakshitha de Silva с соавт. представлены литературные данные, опубликованные в период 1945–2022 гг., включающие описание 30 детей с вальгусной деформацией нижних конечностей, что говорит о редкости данной патологии [27][28]. В работах зарубежных коллег среди поражений опорно-двигательной системы у пациентов встречались: костно-мышечная боль, остеопения, вальгусная деформация конечностей и переломы [5][6][19–21]. В нашем исследовании вальгусная деформация нижних конечностей наблюдалась у 20% пациентов (n=10, ДИ (10, 34)), переломы у 16% пациентов (n=8, ДИ (7; 29)), что в среднем сопоставимо с данными зарубежных исследований [5][6][19–21].

Скелетные нарушения при ПГПТ, такие как вальгусная деформация и переломы, могут встречаться при различных формах рахита, что в свою очередь затрудняет постановку верного диагноза [29–31]. Повышение уровня ПТГ при таких заболеваниях, как Х-сцепленный гипофосфатемический или витамин-Д-зависимый рахит, носит вторичный характер, в связи с чем необходимо проводить тщательную дифференциальную диагностику на основании клинико-лабораторных параметров [34].

Среди неврологических проявлений при ПГПТ наблюдаются утомляемость, головная боль, в редких случаях встречаются депрессия и галлюцинации. Утомляемость является неспецифичным симптомом ПГПТ и по результатам нашего исследования наблюдалась у 36% пациентов с разной степенью гиперкальциемии, что соответствует данным зарубежных исследований [6][17][19][33]. Депрессия, как симптом ПГПТ у детей, описана в единичных исследованиях [33][34] и была выявлена у 1 нашего пациента с умеренной гиперкальциемией. Пациент длительно наблюдался у психиатра, и лишь через 2 года был диагностирован ПГПТ. После оперативного лечения симптомы депрессии не наблюдались. По данным литературы, тяжесть нейропсихологических жалоб не всегда коррелирует со степенью гиперкальциемии, однако с увеличением концентрации кальция повышается риск развития острого психоза [36].

В проведенном нами исследовании у 2 пациентов единственными жалобами были чувство сдавления в области шеи и кашель, послужившие поводом для дальнейшего обследования. У одного пациента дискомфорт в области шеи наблюдался при смене положения тела. В ходе обследования, помимо образования ОЩЖ, выявлен узел щитовидной железы. Проведена паратиреоидэктомия левой нижней ОЩЖ, по результатам морфологического исследования выявлена аденома размером 2,5х0,7х0,7 см. У второго пациента образование ОЩЖ располагалось интратиреоидно. Проведена гемитиреоидэктомия справа, по результатам морфологического исследования выявлена интратиреоидная аденома ОЩЖ диаметром 0,8 см. Вероятно, наличие дополнительного образования в щитовидной железе у одного пациента и атипическое расположение образования ОЩЖ у другого в совокупности послужило появлению симптомов кашля и чувства сдавления в области шеи. После оперативного лечения данные жалобы не возникали.

В исследовании у 2 пациентов на фоне гиперкальциемии наблюдалось повышение температуры до субфебрильных значений при отсутствии признаков воспаления. На сегодняшний день нет официальных данных о том, что повышение уровня кальция способствует развитию гипертермии. В зарубежной литературе имеются единичные описания гипертермии у взрослых с ПГПТ [37][38]. У пациентов были исключены хронические инфекции и другие воспалительные процессы. По описанию авторов, после проведенной паратиреоидэктомии наблюдалось снижение температуры тела [35][36], что также отмечалось и у наших пациентов. Вероятно, это является исключительным признаком у конкретных пациентов, вызванной индивидуальной чувствительностью центра терморегуляции на повышение кальция крови.

По утверждению некоторых авторов, тяжесть клинических симптомов при ПГПТ, вероятно, связана с более значительным повышением кальция крови [13]. Однако ряд пациентов с легкой степенью гиперкальциемии могут иметь тяжелые проявления ПГПТ, в том числе МКБ и деформацию нижних конечностей, и наоборот, часть пациентов с тяжелой гиперкальциемией могут предъявлять жалобы лишь на симптомы диспепсии и утомляемость (табл. 4). В работе Deva Boone с соавт. представлены данные 20 081 взрослого пациента с ПГПТ [14]. В зависимости от уровня кальция, пациенты были разделены на 2 группы: показатели в пределах 10–11 мг/дл (2,5–2,75 ммоль/л) соответствовали первой группе, >11 мг/дл (>2,75 ммоль/л) — второй. По результатам анализа было показано, что субъективные жалобы и осложнения ПГПТ могут возникать при всех уровнях кальция, даже при незначительном его повышении [14]. Подобные результаты были получены в ретроспективном исследовании Huan Yan с соавт. с участием 2266 взрослых пациентов с ПГПТ [15]. Пациенты были разделены на 3 группы по уровню кальция, среди которых 303 человека имели нормокальциемический вариант ПГПТ. При анализе данных между наличием симптомов и уровнем кальция крови прямой корреляции выявлено не было.

По результатам нашего исследования, статистически значимой связи между наличием отдельных клинических проявлений ПГПТ и степенью гиперкальциемии выявлено не было, однако отмечена статистическая тенденция наличия отдельных проявлений заболевания (гиперкальциурия, снижение массы тела, рвота, боль в животе, запоры, эзофагит, боль в ребрах, нарушение походки) с увеличением уровня кальция крови, а также была выявлена положительная ассоциация между гиперкальциурией и гиперкальциемией. Установлено, что у пациентов с тяжелой гиперкальциемией количество клинических признаков значимо выше, чем у пациентов с легкой или умеренной степенью гиперкальциемии.

## ОГРАНИЧЕНИЯ ИССЛЕДОВАНИЯ

В ходе исследования могли возникнуть смещения результатов по причине недостаточного объема выборки в связи с низкой встречаемостью заболевания. Распределение пациентов по группам по степени гиперкальциемии проводилось без учета коррекции кальция по альбумину.

## ЗАКЛЮЧЕНИЕ

Клинические проявления ПГПТ у детей разнообразны, в связи с чем пациенты могут наблюдаться у врачей разных специальностей — педиатров, гастроэнтерологов, нефрологов, неврологов, травматологов-ортопедов.

Тяжесть клинических симптомов не всегда соответствует уровню кальция крови: у пациентов с легкой гиперкальциемией могут наблюдаться поражения ЖКТ, МКБ, деформация конечностей, а некоторые пациенты с умеренной или тяжелой гиперкальциемией могут иметь жалобы лишь на диспепсию и утомляемость.

При наличии таких симптомов и заболеваний, как утомляемость, тошнота, боль в ногах, деформация нижних конечностей, низкотравматические переломы, мочекаменная болезнь, гастрит, необходимо исследовать уровень кальция в крови у детей.

## ДОПОЛНИТЕЛЬНАЯ ИНФОРМАЦИЯ

Источники финансирования. Работа выполнена по инициативе авторов без привлечения финансирования.

Конфликт интересов. Авторы декларируют отсутствие конфликта интересов.

Участие авторов. Бенина А.Р., Колодкина А.А. — поисково-аналитическая работа и подготовка финальной версии статьи; Колодкина А.А., Калинченко Н.Ю., Безлепкина О.Б. — редактирование текста, внесение ценных замечаний. Все авторы одобрили финальную версию статьи перед публикацией, выразили согласие нести ответственность за все аспекты работы, подразумевающую надлежащее изучение и решение вопросов, связанных с точностью или добросовестностью любой части работы.
